# Differential Expression Profiles of the Transcriptome and miRNA Interactome in Synovial Fibroblasts of Rheumatoid Arthritis Revealed by Next Generation Sequencing

**DOI:** 10.3390/diagnostics9030098

**Published:** 2019-08-18

**Authors:** Chia-Chun Tseng, Ling-Yu Wu, Wen-Chan Tsai, Tsan-Teng Ou, Cheng-Chin Wu, Wan-Yu Sung, Po-Lin Kuo, Jeng-Hsien Yen

**Affiliations:** 1 Graduate Institute of Clinical Medicine, College of Medicine, Kaohsiung Medical University, Kaohsiung 80708, Taiwan; 2 Department of Internal Medicine, Kaohsiung Municipal Ta-Tung Hospital, Kaohsiung 80145, Taiwan; 3 Division of Rheumatology, Department of Internal Medicine, Kaohsiung Medical University Hospital, Kaohsiung 80708, Taiwan; 4 Institute of Biomedical Science, National Sun Yat-Sen University, Kaohsiung 80424, Taiwan

**Keywords:** rheumatoid arthritis, miRNA, synovial fibroblast

## Abstract

Using next-generation sequencing to decipher the molecular mechanisms underlying aberrant rheumatoid arthritis synovial fibroblasts (RASF) activation, we performed transcriptome-wide RNA-seq and small RNA-seq on synovial fibroblasts from rheumatoid arthritis (RA) subject and normal donor. Differential expression of mRNA and miRNA was integrated with interaction analysis, functional annotation, regulatory network mapping and experimentally verified miRNA–target interaction data, further validated with microarray expression profiles. In this study, 3049 upregulated mRNA and 3552 downregulated mRNA, together with 50 upregulated miRNA and 35 downregulated miRNA in RASF were identified. Interaction analysis highlighted contribution of miRNA to altered transcriptome. Functional annotation revealed metabolic deregulation and oncogenic signatures of RASF. Regulatory network mapping identified downregulated FOXO1 as master transcription factor resulting in altered transcriptome of RASF. Differential expression in three miRNA and corresponding targets (hsa-miR-31-5p:WASF3, hsa-miR-132-3p:RB1, hsa-miR-29c-3p:COL1A1) were also validated. The interactions of these three miRNA–target genes were experimentally validated with past literature. Our transcriptomic and miRNA interactomic investigation identified gene signatures associated with RASF and revealed the involvement of transcription factors and miRNA in an altered transcriptome. These findings help facilitate our understanding of RA with the hope of serving as a springboard for further discoveries relating to the disease.

## 1. Introduction

Rheumatoid arthritis (RA) is an autoimmune disease characterized by sustained chronic inflammation resulting in joint damage and severe disability. New and more effective therapies developed over the past two decades targeting the inflammatory mediators and immune cells [1,2] have revolutionized the management of RA [3]. However, disease remission remains uncommon [4]. Furthermore, therapies targeting different aspects of the immune response render patients more susceptible to infection [5]. In light of these challenges, approaches that focus on other components of joint inflammation have been proposed as potential solutions [4].

In RA, inflammatory cytokines, such as interleukin (IL)-6 and tumor necrosis factor-alpha (TNF-α), cause dysregulated proliferation and drive a migratory and invasive phenotype of synovial fibroblast [6,7] (also known as fibroblast-like synoviocytes (FLS)) [8], resulting in pannus formation. Synovial fibroblasts are directly involved in cartilage and bone destruction by production of matrix metalloproteinases (MMPs) and activation of osteoclasts through receptor activator of nuclear factor kappa-B ligand (RANKL) [8]. Synovial fibroblasts also contribute to inflammatory amplification via IL-6 production [9]. Although immune cells and inflammatory mediators have been feasible targets for RA treatment [1,2,10], there has never been a treatment strategy targeting specifically the aggressiveness of synovial fibroblasts. Compared with inflammatory mediators and immune cells, little is known about the causes of the particularly aggressive nature of RA synovial fibroblasts. Therefore, a more comprehensive understanding of synovial fibroblasts in RA may yield promising novel therapeutic targets.

A substantial number of epigenetic mechanisms are known to regulate dynamic changes in gene expression of various cells. MicroRNA (miRNA) is one best-known mechanism which plays a central role in post-transcriptional modification of gene expression via antisense binding to messenger RNA (mRNA). It was well-established that miRNA participated in pathophysiological processes of various autoimmune diseases, such as ankylosing spondylitis and uveitis [11,12]. However, the roles of miRNA in RA synovial fibroblasts were less clear.

In the past, several studies explored transcriptome of RA synovial fibroblasts utilizing RNA-seq [13,14]. However, they compared transcriptome changes before and after inflammatory mediator stimulation rather than transcriptome differences between RA and normal donors. Moreover, whether and how miRNA contributed to transcriptome changes in RA was not fully understood. To explore the full mRNA transcriptome and miRNA interactome of RA synovial fibroblasts in genome-wide scale, we performed transcriptome-wide RNA-seq and small RNA-seq in synovial fibroblasts from RA patient and normal donor to reveal changes to the synovial fibroblasts transcriptome and to elucidate the contributing miRNA to transcription signatures in RA synovial fibroblasts. We further applied interaction analysis of transcription factors, miRNA, and target genes, functional annotation and regulatory network mapping to elucidate involved molecular programs. Experimentally validated targets of miRNA and data from publicly available databases were integrated to validate our results. These results offer a map to the synovial fibroblasts transcriptome and miRNA interactome and shed light on the pathophysiology of RA.

## 2. Methods

### 2.1. Synovial Fibroblasts

Primary human synovial fibroblasts isolated from one normal donor and one RA subject were obtained from Cell Applications Inc (San Diego, CA, USA). Synovial fibroblasts were cultured with Synoviocyte Growth Medium (Cell Applications Inc. San Diego, CA, USA) at 37 °C and 5% CO_2_. When the cells had grown to confluence, the normal synovial fibroblasts and RA synovial fibroblasts were harvested for total RNA extraction and transcriptome profiling. First passage synovial fibroblasts were used for this study to avoid the influence of repeated passages and accumulation of chromosomal aberrations, which may influence gene expression. The study was exempted from the institutional review board requirement because the research utilized de-identified human cells obtained from commercial entities.

### 2.2. RNA Extraction

RNA was isolated from primary cultured synovial fibroblasts with the Trizol reagent (Invitrogen, Carlsbad, CA, USA) for generation of both long and small RNA libraries for sequencing. The RNA quantity and quality were evaluated using the ND-1000 spectrophotometer (Nanodrop Technology, Wilmington, DE, USA) and the Agilent RNA 6000 labchip kit with Agilent 2100 Bioanalyzer instrument (Agilent Technologies Inc, Santa Clara, CA, USA), respectively. The qualities of extracted RNA were shown in Table S1.

### 2.3. RNA-Seq

Agilent’s SureSelect Strand Specific RNA Library Preparation Kit was used for library construction. Briefly, RNA was purified and fragmented using poly-T oligo-attached magnetic beads followed by complementary DNA (cDNA) strand synthesis. Next, cDNA 3′ ends were adenylated and adapters ligated followed by library amplification. The libraries were size-selected using AMPure XP Beads (Beckman Coulter). The sequence was directly determined using sequencing-by-synthesis technology on Illumina/Solexa platform (150 paired-end cycles) via the TruSeq SBS Kit. Raw sequences were obtained from the Illumina Pipeline software bcl2fastq v2.0 to generate total reads of 9G per sample. For RNA-seq analysis, initially the sequences generated went through a filtering process to obtain qualified reads. Trimmomatic was implemented to trim or remove the reads according to the quality score. HISAT2 was used for mapping and alignment to reference human genomes, based on hierarchical graph FM index (GFM index) [15]. The gene expression level was calculated as Fragments Per Kilobase of transcript per Million mapped reads (FPKM). Further differential expression analysis was performed based on cuffdiff (cufflinks version 2.2.1) with genome bias detection/correction [16].

### 2.4. Small RNA-Seq

The Illumina TruSeq Small RNA sample preparation protocol was used to produce the small RNA-seq libraries from total RNA. Briefly, total RNA were ligated with 3′ and 5′ adaptors and reverse transcribed followed by polymerase chain reaction (PCR) amplification to obtain cDNA constructs. The enriched cDNA constructs were size-fractionated and purified on a 6% polyacrylamide gel electrophoresis with the bands containing the 18–40 nucleotide RNA fragments (140–155 nucleotides in length with both adapters). Then, 75 bp single-end sequencing was performed on Illumina/Solexa platform with total reads of 10M per sample. Sequencing data was processed with the Illumina software. We used miRDeep2 to analyze the generated raw data, evaluate the overall sequencing qualities and determine the miRNA expression profiles. For expression analysis of miRNA, the gene expression level was calculated as Reads Per Million (RPM).

### 2.5. RNA-Seq Analysis

**Figure 1:**
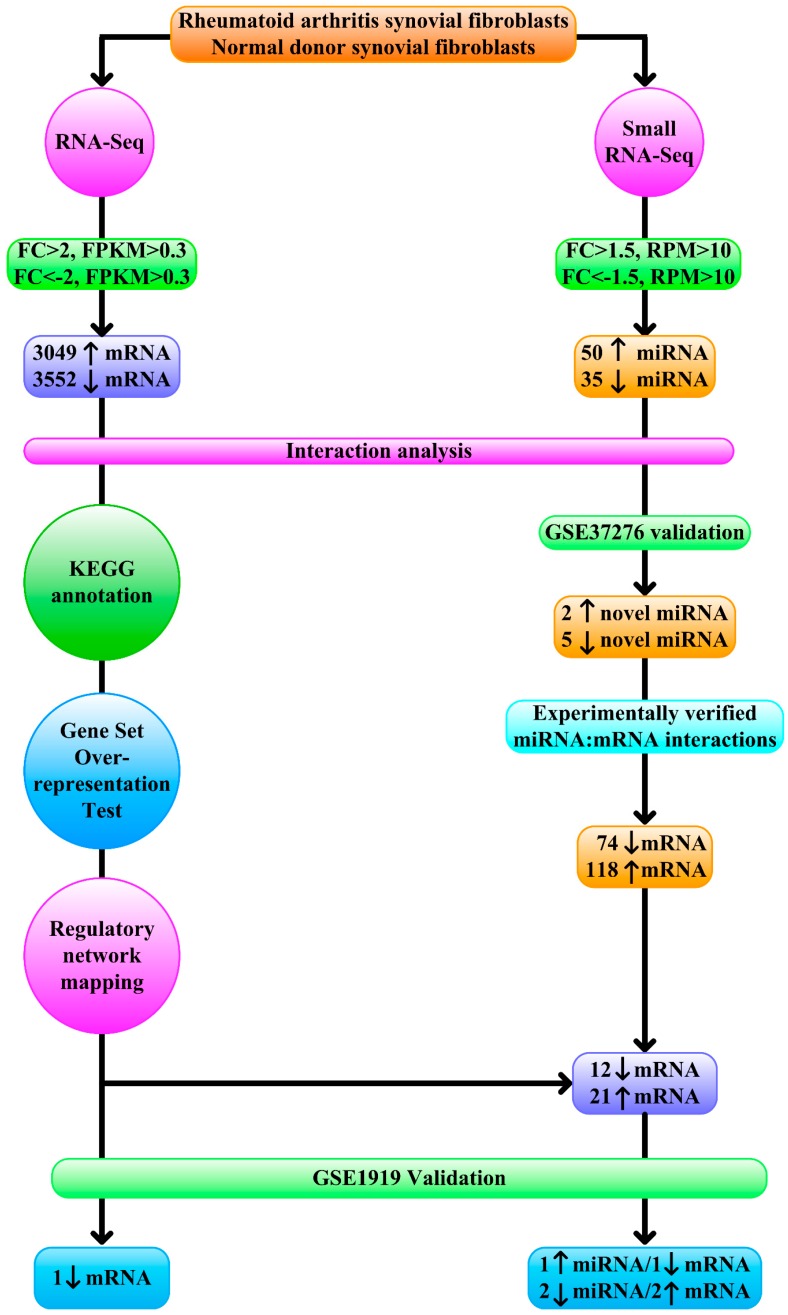
Schematic representation of the next-generation sequencing data analytical workflow. 3049 upregulated (fold change > 2, Fragments Per Kilobase of transcript per Million (FPKM) > 0.3), 3552 downregulated protein-coding mRNA (fold change < −2, FPKM > 0.3), 50 upregulated (fold change > 1.5, Reads Per Million (RPM) > 10), and 35 downregulated (fold change < −1.5, RPM > 10) miRNA were examined with interaction analysis. Kyoto Encyclopedia of Genes and Genomes (KEGG) annotation, Gene Set Overrepresentation Test, regulatory network mapping, and mRNA validation with GSE1919 identified one downregulated mRNA as master regulator. Seven novel miRNAs were also verified with GSE37276. Of 74 downregulated and 118 upregulated mRNA as experimentally verified targets of validated novel miRNA, 12 and 21 displayed concordant changes in RNA-seq, and one downregulated and two upregulated mRNA were validated with GSE1919.

To identify differentially expressed mRNA from RNA-seq data, we adopted previous protocols [17]. Briefly, we retrieved mRNA with a minimum absolute fold change of 2 [17] and FPKM > 0.3 in RA or normal donor (Figure 1), since a threshold FPKM value of 0.3 balanced the numbers of false positives and false negatives [18].

### 2.6. Small RNA-Seq Analysis

In small RNA-seq analysis, we set the criteria of differential expression based on past studies [19,20]. To identify differentially expressed miRNA, we retrieved miRNA with a minimum absolute fold change of 1.5 [19] and RPM > 10 in RA or normal donor, since a threshold of 10 RPM identified all functional miRNAs while removing many inconsequential reads [20] (Figure 1).

### 2.7. Interaction Analysis of Transcription Factors, miRNA, and Target Genes

Taking advantage of TFmiR [21], differentially expressed protein-coding mRNA and miRNA and interactions between them were tested for overrepresentation of RA-specific networks and significance of transcription factor–target gene interaction and miRNA–target gene interaction (Figure 1). TFmiR utilized information provided by established regulatory databases of experimentally validated interactions and scientific literature to facilitate research on transcriptional and post-transcriptional interactions between transcription factors, miRNA and target genes. The significance of transcriptional and post-transcriptional regulatory interactions from deregulated mRNA and miRNA was assessed using the hypergeometric distribution test. It also assessed relevance of the provided deregulated inputs to the disease-associated genes/miRNA.

### 2.8. Transcript Functional Annotation

To better appreciate the functional picture of differentially expressed mRNA, we employed Kyoto Encyclopedia of Genes and Genomes (KEGG) database [22] to generate functional maps of differentially expressed mRNA (Figure 1). Gene Set Overrepresentation Test was executed with PANTHER [23] to yield insights into biological pathways involved in RA (Figure 1).

### 2.9. Regulatory Network Mapping

For discovery of enriched transcription factors and their targets in the RNA-seq data, we executed iRegulon [24] that utilized ChIP-Seq and motif occurrence information to elucidate master transcription factors driving differentially expressed genes observed in RA synovial fibroblasts (Figure 1). The identified master transcription factors were validated with the Gene Expression Omnibus (GEO) dataset GSE1919 [25] to confirm differential expression of identified master transcription factor (Figure 1). The Gene Multiple Association Network Integration Algorithm (GeneMANIA) was queried to investigate the most related genes to reconstruct regulatory networks from validated master transcription factors [26].

### 2.10. miRNA Profile Validation

To confirm differential expression of miRNA between RA synovial fibroblasts and normal fibroblasts, we retrieved expression profiles of miRNA in RA synovial fibroblasts and normal fibroblasts from GSE37276 [27] (Figure 1). Validated differentially expressed miRNAs were reserved for further investigation.

### 2.11. Experimentally Validated Target*s* of Validated Differentially Expressed miRNA

To identify experimentally validated target*s* of differentially expressed miRNA, we downloaded target*s* of differentially expressed miRNA with strong experimental evidence (reporter assay or Western blot) from miRTarBase [28] (Figure 1). Overlap of differentially expressed mRNA in RNA-seq data and experimentally validated target*s* of validated differentially expressed miRNA were compared with the GEO dataset GSE1919 [25] to confirm differential expression of targets of validated differentially expressed miRNA (Figure 1).

### 2.12. Statistical Analysis

For the Gene Set Overrepresentation Test, pathways with a false discovery rate less than 0.05 were considered significantly enriched. During validation with GEO dataset, expression profiles obtained from GEO dataset [25,27] were analyzed with GEO2R [29]. We intersected genes with differential expression in RNA-seq/small RNA-seq with probes of GEO dataset. P values less than 0.05 in GEO dataset were considered validation in GEO dataset.

## 3. Results

### 3.1. Contribution of Transcription Factors and miRNA to Differentially Expressed mRNA

**Figure 2:**
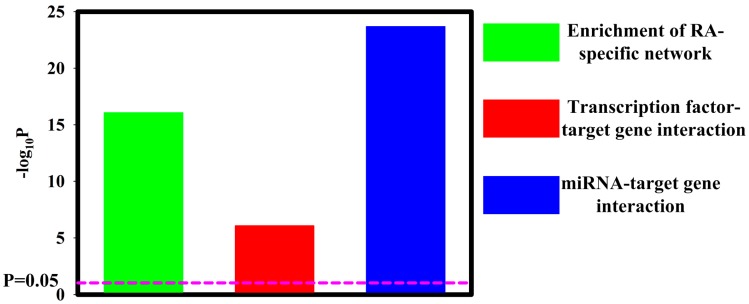
Interaction analysis of transcription factors, miRNA, and target genes. TFmiR analysis revealed significant enrichment of RA-specific network (*p* = 8.48 × 10^−17^). Significant transcription factor–target gene interaction (8.30 × 10^−7^) and miRNA–target gene interaction (2.03 × 10^−24^) were revealed.

In the RNA-seq analysis (Figure 1), we identified a total of 6601 differentially expressed protein-coding mRNA-3049 upregulated mRNA and 3552 downregulated mRNA (Figure S1, Figure S2A). The details of upregulated mRNA and downregulated mRNA were shown in Tables S2 and S3. In the small RNA-seq analysis (Figure 1), 50 upregulated miRNA and 35 downregulated miRNAs were discovered (Figure S1, Figure S2B). The details of upregulated miRNA and downregulated miRNA are shown in Tables S4 and S5. As expected, significant overlaps of dysregulated miRNA/mRNA with RA-specific network were noted (*p* = 8.48 × 10^−17^) (Figure 2). This observation indicated a contribution of dysregulated miRNA/mRNA to RA development. Additionally, transcription factors and dysregulated miRNA in RA synovial fibroblasts both contributed significantly to altered mRNA expression (*p* = 8.30 × 10^−7^ and *p* = 2.03 × 10^−24^, respectively) (Figure 2).

### 3.2. Functional Annotation Differentially Expressed mRNA

To better understand the molecular functions associated with differentially expressed mRNA, we utilized KEGG database to perform function annotation (Figure 1). Interestingly, the top-ranking category associated with both upregulated and downregulated mRNA was metabolic pathway (Figure S3). Since tumor cells are well documented to rewire their metabolism and energy production networks to support and enable rapid proliferation, continuous growth, survival in harsh conditions, invasion, metastasis and resistance to cancer treatments [30], these metabolic changes might be necessary to cater to demands of sustained inflammation and aggressive natures in RA synovial fibroblasts.

Further analysis with Gene Set Overrepresentation Test for upregulated mRNA (Figure 1) identified several proliferation and migration related pathways enriched in upregulated genes, including cell division, mitotic cell cycle checkpoint, mitotic cell cycle phase, mitotic cell cycle phase transition, regulation of cell cycle activity and regulation of epithelial cell migration (Figure S4). In contrast, these proliferation and migration-related pathways were not associated with downregulated genes (Figure S5) in Gene Set Overrepresentation Test for downregulated mRNA (Figure 1). The data collectively supported previous observations that RA synovial exhibited numerous tumor-like characteristics with excessive proliferation, migration and invasion [31,32,33].

### 3.3. Regulatory Network Mapping

**Figure 3:**
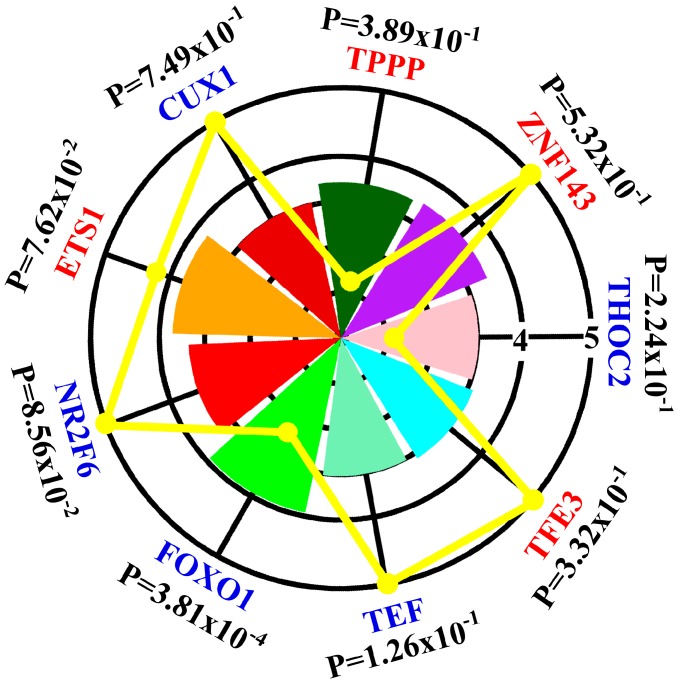
Graphical representation of the iRegulon analysis results. Each chart slice corresponded to a transcription factor identified by iRegulon whose radius was associated normalized enrichment score. The color of the transcription factor indicated upregulation (red) or downregulation (blue) in RNA-seq data and the yellow line showed corresponding absolute values of fold change in RNA-seq data. P values indicated results of validation with GSE1919. For example, FOXO1 is one transcription factor identified by iRegulon which was downregulated (blue color) with fold change –2.38 (yellow line) in RNA-seq and normalized enrichment score 3.92 (green slice). Validation with GSE1919 yielded P value of 3.81 × 10^−4^.

**Figure 4:**
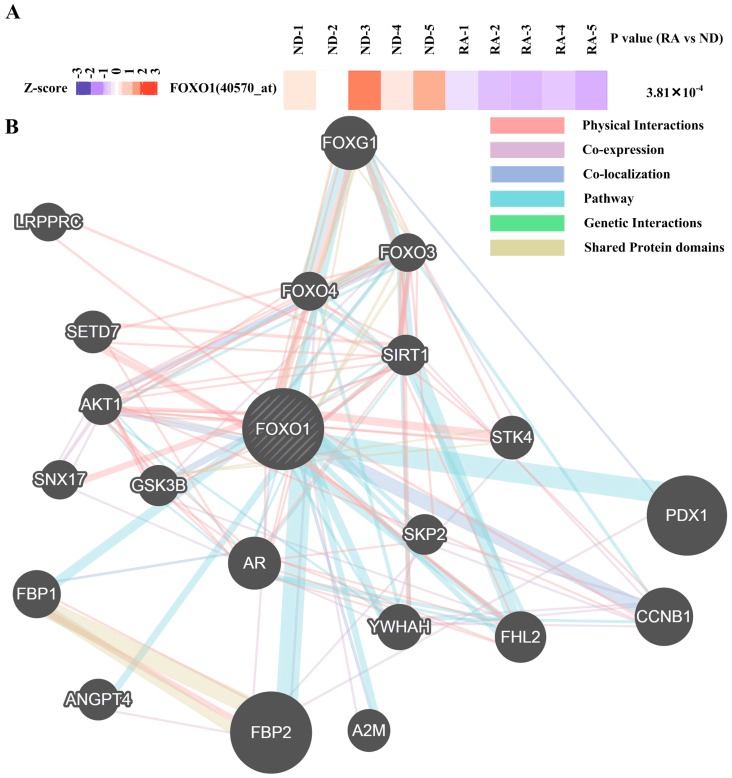
Expression and gene interactions of FOXO. (**A**) Heatmap showing the scaled expression of FOXO1 in rheumatoid arthritis (RA) synovial fibroblasts and normal donor (ND) synovial fibroblasts as well as P values in GSE1919 with corresponding probe identification shown in parentheses. (**B**) Gene interaction of FOXO1 with other proteins visualized by Gene Multiple Association Network Integration Algorithm (GeneMANIA) was shown.

To gain insight in the spectrum of transcription factors that could drive the altered mRNA transcriptome in RA synovial fibroblasts, we used iRegulon [24] to map the transcription factors predicted to act as upstream regulators for upregulated and downregulated mRNA (Figure 1). Nine transcription factors whose expression in RA synovial fibroblasts and normal donor synovial fibroblasts not previously studied were predicted to act as upstream regulators (Figure 3). One transcription factor (FOXO1) showed differential expression in both RNA-seq data (Figure S2A, Figure 3) and GEO dataset GSE1919 [25] (Figure 4A). Gene interaction of FOXO1 with other proteins visualized by GeneMANIA is shown in Figure 4B.

### 3.4. Validation of Differentially Expressed miRNA

**Figure 5:**
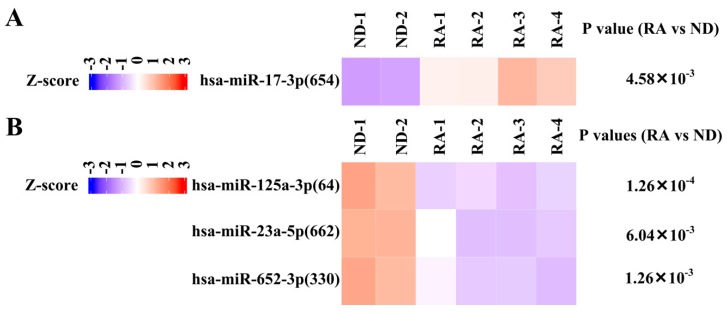
Expression of hsa-miR-17-3p, hsa-miR-125a-3p, hsa-miR-23a-5p, and hsa-miR-652-3p in Gene Expression Omnibus (GEO) dataset. (**A**) Heatmap showing the scaled expression of upregulated miRNA (hsa-miR-17-3p) in rheumatoid arthritis (RA) synovial fibroblasts compared with normal donor (ND) synovial fibroblasts as well as P value in GSE37276 with probe identification shown in parentheses. (**B**) Heatmap showing the scaled expression of downregulated miRNA (hsa-miR-125-3p, hsa-miR-23a-5p, hsa-miR-652-3p) as well as P values in GSE37276 with probe identification shown in parentheses.

**Figure 6:**
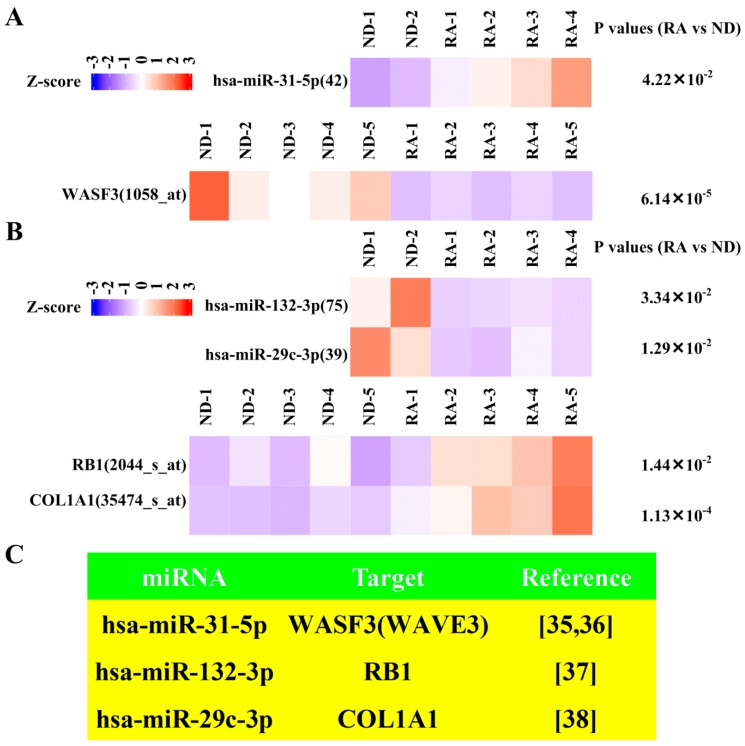
Expression of miRNA (hsa-miR-31-5p, hsa-miR-132-3p, hsa-miR-29c-3p) and corresponding targets (WASF3, RB1, COL1A1) in GEO dataset. (**A**) Heatmap showing the scaled expression of upregulated miRNA (hsa-miR-31-5p) and downregulated corresponding targets (WASF3) in rheumatoid arthritis (RA) synovial fibroblasts compared with normal donor (ND) synovial fibroblasts as well as P values in GSE1919 and GSE37276 with probe identification shown in parentheses. (**B**) Heatmap showing the scaled expression of downregulated miRNA (hsa-miR-132-3p, hsa-miR-29c-3p) and upregulated corresponding targets (RB1, COL1A1) in RA and ND synovial fibroblasts as well as P values in GSE1919 and GSE37276 with probe identification shown in parentheses. (**C**) Literature supporting interactions of miRNA and corresponding targets.

To confirm differential expression of miRNA in RA synovial fibroblast, we compared our small RNA-seq results with miRNA profiling of GSE37276 [27] (Figure 1, Figure S1). Although eight miRNAs were identified, the role of only one miRNA (hsa-miR-146a-5p) in RA synovial fibroblast has been well-studied [34]. Thus, we focus on remaining two upregulated miRNA and five downregulated miRNAs (Figure S2B, Figure 5, Figure 6A,B).

### 3.5. Dysregulated Targets of Validated Upregulated/Downregulated miRNA

To reveal dysregulated targets of validated upregulated/downregulated miRNA, we first retrieved experimentally verified targets of validated miRNA from miRTarBase (Figure 1). In total, 74 experimentally verified targets of validated upregulated miRNA and 118 experimentally verified targets of validated downregulated miRNA were obtained (Figure S1). After intersection with downregulated mRNA and upregulated mRNA from RNA-seq, 21 upregulated mRNA and 12 downregulated mRNA were revealed (Figure S1). After validation with GSE1919 [25] (Figure 1), one downregulated mRNA (Figure S2A, Figure 6A) and two upregulated mRNA in RNA-seq (Figure S2A, Figure 6B) were singled out. The literature supporting the interaction between these three pairs of miRNA–target genes (hsa-miR-31-5p:WASF3, also called WAVE3, hsa-miR-132-3p:RB1, hsa-miR-29c-3p:COL1A1) [35,36,37,38] are shown in Figure 6C.

## 4. Discussion

RA synovial fibroblasts constitute a unique cell type that distinguishes RA from other arthritic conditions and contribute significantly to the initiation and perpetuation of the disease [39,40]. Thus a detailed understanding of the internal state of synovial fibroblasts in RA pathogenesis is critical. By combining RNA-seq and small RNA-seq data, this study provides a global view of the transcriptome and miRNA interactome profile and discloses the significant contribution to altered transcriptome by miRNA in RA synovial fibroblasts. Additionally, we identified and validated one transcription factor (FOXO1), which contributed to altered transcriptome in RA synovial fibroblasts utilizing iRegulon and past microarray results [25]. Moreover, three pairs of miRNA–target gene interaction (hsa-miR-31-5p:WASF3, hsa-miR-132-3p:RB1, hsa-miR-29c-3p:COL1A1) were validated through combining small RNA-seq with RNA-seq results, miRTarBase, and previous mRNA/miRNA profiling studies [25,27]. These results highlight the particular roles played by these transcription factors and miRNA in RA synovial fibroblasts.

Through interaction analysis of miRNA and target genes, a significant contribution to transcriptome alteration by miRNA was revealed. This exemplifies the complexity of interaction between miRNA and mRNA in RA synovial fibroblasts. This miRNA interactome provides a comprehensive molecular basis for additional information on the pathogenetic mechanisms of rheumatoid arthritis and offer a roadmap to directly probe miRNAs of interest with their likely downstream signaling pathways and functional roles of target proteins. Furthermore, miRNAs target multiple mRNAs in a network and, via dysregulation, implicated in numerous autoimmune diseases [41]. Although targets of several miRNA (hsa-miR-17-3p, hsa-miR-125a-3p, hsa-miR-23a-5p, and hsa-miR-652-3p) (Table S6) failed to be validated with published data, significant evidence of interaction between these targets and corresponding miRNA was found from miRTarBase, and these targets should be followed up in future studies. Moreover, considering the successful application of antisense oligonucleotide strategies in human diseases [42] and therapeutic potential of miRNA in preclinical studies of RA [43], targeting such a dysfunctional miRNA–mRNA interaction may hold promise for RA.

When we annotated differentially expressed transcripts using KEGG database, metabolic pathway was the top-ranking functional category. Several studies demonstrated that the dysregulated synovial cellular bioenergetics switched RA synovial fibroblast profiles and promoted inflammatory natures of RA synovial fibroblasts [44,45]. Furthermore, rewiring of synovial fibroblasts metabolism facilitated resolution of arthritis in the animal model [44,45]. Combined with beneficial effects of metabolic reprogramming in other autoimmune diseases clinically [46], our study draws attention to the therapeutic potential of metabolic reprogramming for RA.

**Figure 7:**
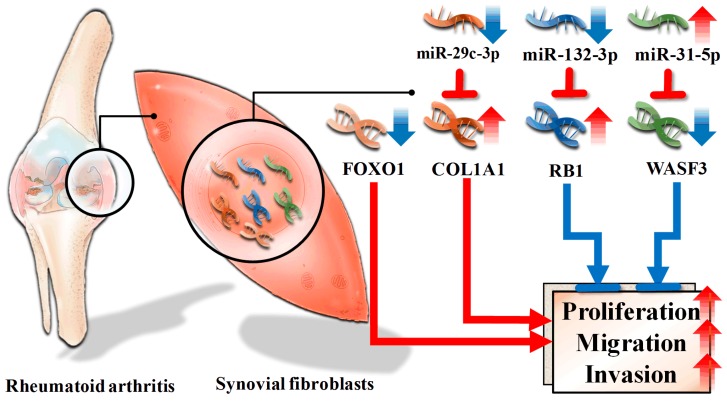
Proposed effects of dysregulated miRNA and transcription factor in rheumatoid arthritis synovial fibroblasts. Downregulated miR-29c-3p resulted in increased COL1A1, together with downregulated FOXO1, increased proliferation, migration, and invasion in rheumatoid arthritis synovial fibroblasts. On the contrary, downregulated miR-132-3p and upregulated miR-31-5p, which contributed to increased RB1 and depressed WASF3, respectively, diminished proliferation, migration, and invasion in rheumatoid arthritis synovial fibroblasts. Inverted T-shape: suppression, blue arrowhead: decrease, red arrowhead: increase, blue arrow: enhancement red arrows: inhibition.

In the step of regulatory network mapping, downregulated FOXO1 was identified as one master regulator. Past studies showed FOXO1 induced apoptosis in RA synovial fibroblasts [47]. Furthermore, differentially expressed genes regulated by FOXO1 (Table S7) also participated in the proliferation, migration, and invasion. For example, CCND1, PAI-1, NOTCH1, ACADM, MEF2C, CDK1, TNNT1, CCNB1, DIO2 facilitated proliferation, migration, and invasion [48,49,50,51,52,53,54,55,56,57,58], while RAB7, RUNX2, ICAM1, NFKB1, TXNIP, IL23A, NEP, catalase, GCK, PPARG, PUMA, AXIN2 suppressed proliferation, migration, and invasion [59,60,61,62,63,64,65,66,67,68,69,70,71]. These results together suggest a contributing role of FOXO1 in RA synovial fibroblast proliferation (Figure 7).

With regard to miRNA, three pairs of verified miRNA–target interaction with validated differential expression of both miRNA and target genes (hsa-miR-31-5p:WASF3, hsa-miR-132-3p:RB1, hsa-miR-29c-3p:COL1A1) were revealed. Hsa-miR-31-5p inhibited proliferation, migration and invasion of malignant cells [72,73]. Furthermore, WASF3 contributed to cancer cells proliferation, migration and invasion [36]. These findings suggest a regulatory role of hsa-miR-31-5p:WASF3 in ameliorating proliferation, migration, and invasion of RA synovial fibroblast (Figure 7). Concerning hsa-miR-132-3p:RB1, it was well-established that hsa-miR-132-3p increased cell proliferation in pancreatic cells [37], and RB1 inhibited malignant cell proliferation [74]. It is possible that downregulated hsa-miR-132-3p acted as a feedback loop to suppress proliferation of RA synovial fibroblasts (Figure 7). Regarding hsa-miR-29c-3p:COL1A1, it appears hsa-miR-29c-3p inhibited proliferation, migration, and invasion of malignant cells [75]. Moreover, COL1A1 promoted proliferation, migration, and invasion of malignancy [76,77]. As a result, downregulation of hsa-miR-29c-3p and upregulation of COL1A1 potentially enhanced proliferation, migration, and invasion of RA synovial fibroblasts (Figure 7).

Conceptually, some of the present data may seem paradoxical, as some differentially expressed miRNA and mRNA resulted in ameliorated disease. However, it was compatible with current knowledge that upregulated negative feedback loop accompanied disease-promoting processes in disease pathogenesis [78]. Similar phenomena involving miRNA and mRNA have been demonstrated in numerous inflammatory diseases and carcinogenesis. For example, miR-10b-5p, which inhibited production of IL-17, the central cytokine of ankylosing spondylitis, was upregulated in ankylosing spondylitis [79]. IGF-I, which rendered cells susceptible to transformation and thereby contributeed to tumor progression, was decreased in breast cancer [80].

In this study, synovial fibroblasts from normal donor rather than osteoarthritis were used for comparison. Most studies of synovial fibroblasts utilized osteoarthritis synovial fibroblasts as controls owing to limited accessibility of normal donor synovial fibroblasts [7,47]. Considering altered transcriptome of osteoarthritis synovial fibroblasts [81], results of this study might be more biologically relevant for RA synovial fibroblasts.

Recent miRNA studies utilized various bioinformatics approaches as screening tools to identify miRNA–mRNA interactome without biological replicates [82,83]. This raised questions about validity of findings from these studies. The identification and validation of miRNA–mRNA target interactions are critical for our understanding of the regulatory networks governing biological processes. In our study, restricting miRNA–mRNA to experimentally validated interactions makes findings of our results more convincing and more suitable to serve as a starting point for investigations of miRNA-based therapies.

## 5. Conclusions

In past decades, advancement in the understanding of molecular bases of disease pathogenesis and the application of new technologies fundamentally transformed our treatment of those diseases [4]. Combining of RNA-seq and small RNA-seq allowed researchers to examine RA synovial fibroblasts at the scale of a complete transcriptome and miRNA interactome. Our analysis identifies transcription factors responsible for an altered transcriptome and elucidates miRNA participating in expression regulation. These findings provide a more comprehensive understanding of the pathophysiology of aberrant synovial fibroblasts activation [39] and the mechanisms underlying the role of synovial fibroblasts in the process leading to RA.

## Supplementary Materials

The following are available online at https://www.mdpi.com/2075-4418/9/3/98/s1.
