# Supplementary material for: Differential Expression Profiles of the Transcriptome and miRNA Interactome in Synovial Fibroblasts of Rheumatoid Arthritis Revealed by Next Generation Sequencing
Source: Diagnostics (Basel). 2019 Aug 18;9(3):98. doi: 10.3390/diagnostics9030098 (PMC6787660; doi:10.3390/diagnostics9030098)
Supplement: Supplementary file 1 [file diagnostics-09-00098-s001.zip › diagnostics-560731-supplementary.docx]

**Table 1.** Next-generation sequencing RNA quality of rheumatoid arthritis synovial fibroblasts and normal donor synovial fibroblasts.

|  | **OD_260_/OD_280_** | **RIN** |
| --- | --- | --- |
| RASF | 1.98 | 9.9 |
| NDSF | 1.95 | 9.9 |

RASF: Rheumatoid arthritis synovial fibroblasts; NDSF: Normal donor synovial fibroblasts; OD: Optical Density; RIN: RNA integrity number.

**Table 7.** Differentially expressed genes regulated by FOXO1 with concordant changes in RNA-seq and literature supporting their regulation by FOXO1.

| **Gene** | **Fold Change** | **Reference** |
| --- | --- | --- |
| CCND1 | 2.47 | [1] |
| PAI-1(SERPINE1) | 3.54 | [2] |
| PITX1 | 4.38 | [3] |
| NOTCH1 | 4.74 | [4] |
| ACADM(MCAD) | 5.37 | [5] |
| MEF2C | 25.48 | [6] |
| IGFALS | >30 | [7] |
| Acyl-CoA oxidase (ACOX1) | >30 | [5] |
| CDK1 | >30 | [8] |
| Troponin T1(TNNT1) | >30 | [9] |
| CCNB1 | >30 | [10] |
| DIO2 | >30 | [11] |
| RAB7A(RAB7) | -2.50 | [12] |
| RUNX2 | -2.93 | [8] |
| CCL2(MCP-1) | -2.96 | [13] |
| ICAM1 | -3.10 | [14] |
| NFKB1 | -3.73 | [14] |
| TXNIP | -5.40 | [15] |
| S1PR4(EDG6) | <-30 | [16] |
| IL23A | <-30 | [16] |
| MME(NEP) | <-30 | [16] |
| Catalase (CAT) | <-30 | [17] |
| GCK(GK) | <-30 | [18] |
| PPARG | <-30 | [19] |
| BBC3(PUMA) | <-30 | [20] |
| AXIN2 | <-30 | [8] |
| CAMKK1 | <-30 | [8] |

The names in parenthesis were aliases of respective genes in the literature.


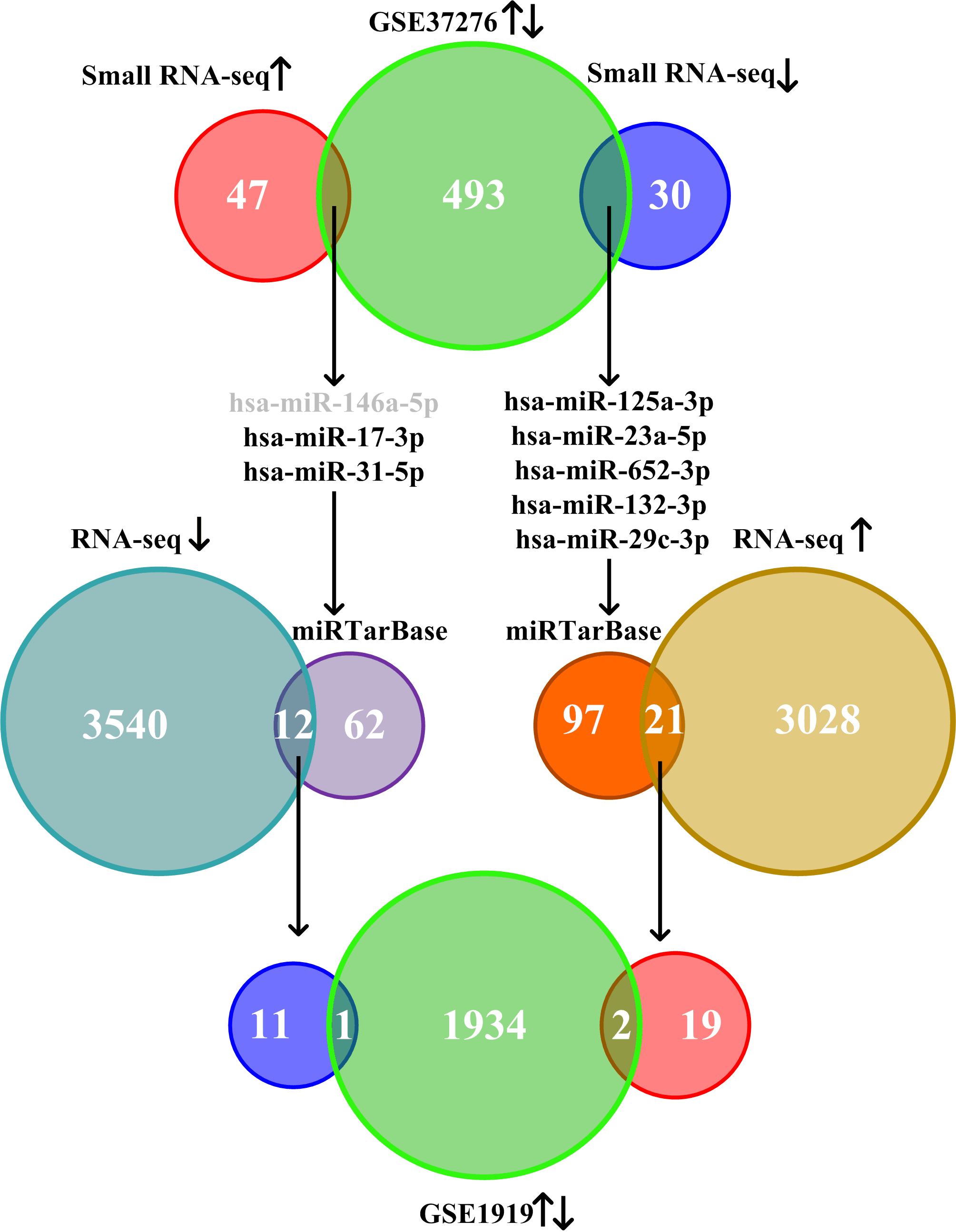


**Figure 1. Overlap of RNA-seq and small RNA-seq results with GSE1919 and GSE37276.** Of 50 upregulated miRNA, three were validated in GSE37276. Of 35 downregulated miRNAs, five were validated in GSE37276. Differential expression of hsa-miR-146a-5p has been studied in RA synovial fibroblasts, thus we focused on remaining seven miRNAs. 74 and 118 mRNA experimentally validated targets of upregulated and downregulated miRNA were retrieved from miRTarBase. After intersection with RNA-seq, 12 and 21 mRNA were identified. Through validation with GSE1919, one downregulated and two upregulated mRNA remained.


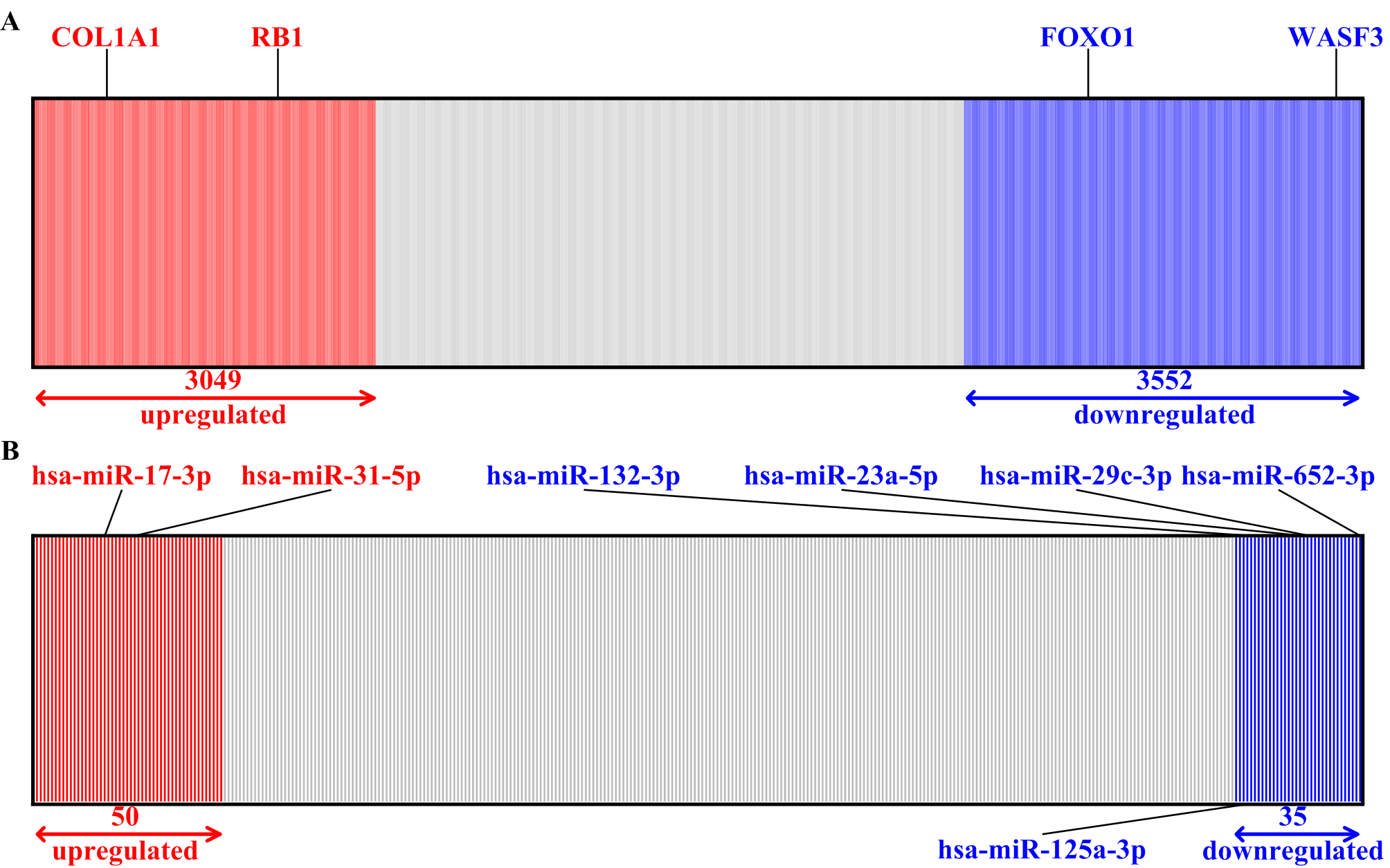


**Figure 2. RNA-seq and small RNA-seq results of synovial fibroblasts.** (a) Graphical representation showing all protein-coding mRNA. Every line was one protein-coding mRNA colored according to corresponding fold change (red: fold change > 2, blue: fold change < -2). mRNA validated in following analysis (COL1A1, RB1, FOXO1, WASF3) were highlighted. (b) Graphical representation showing all miRNA. Every line was one miRNA colored according to corresponding fold change (red: fold change > 1.5, blue: fold change < -1.5). miRNA validated in following analysis (hsa-miR-17-3p, hsa-miR-31-5p, hsa-miR-125a-3p, hsa-miR-132-3p, hsa-miR-23a-5p, hsa-miR-29c-3p, hsa-miR-652-3p) were highlighted.


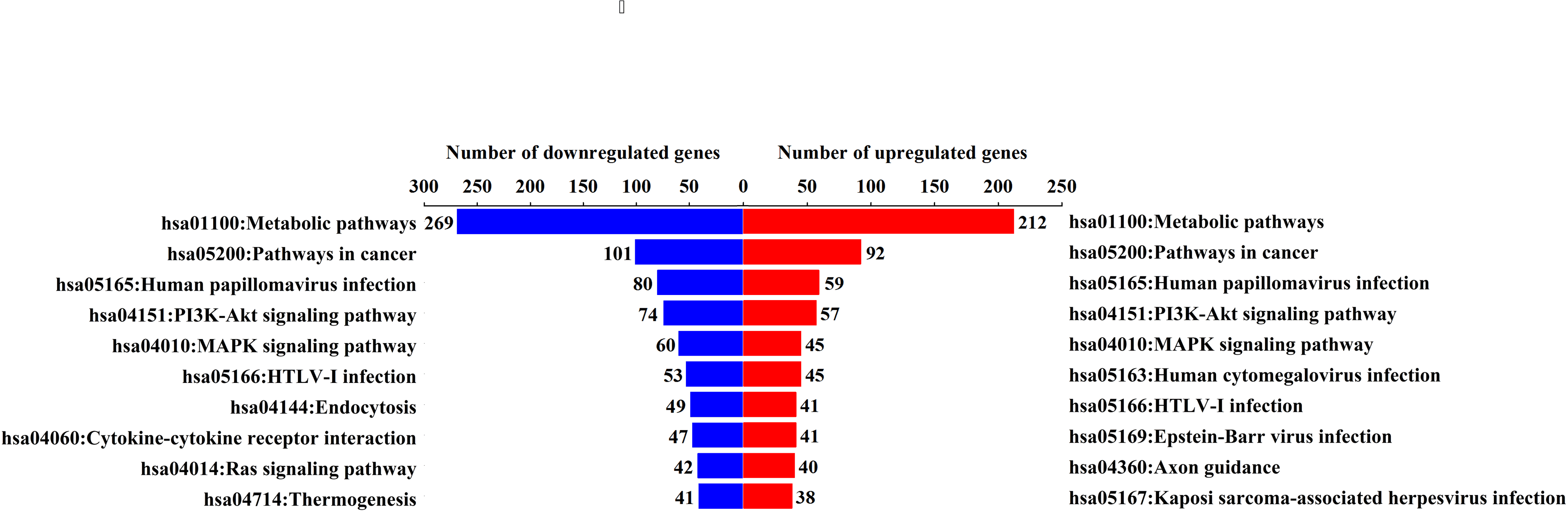


**Figure 3. Functional annotation of differentially expressed genes.** Functional annotation of upregulated genes (red bar) and downregulated genes (blue bar) were listed*.* The numbers accompanying the bars were the total numbers of genes that were mapped to each functional category.


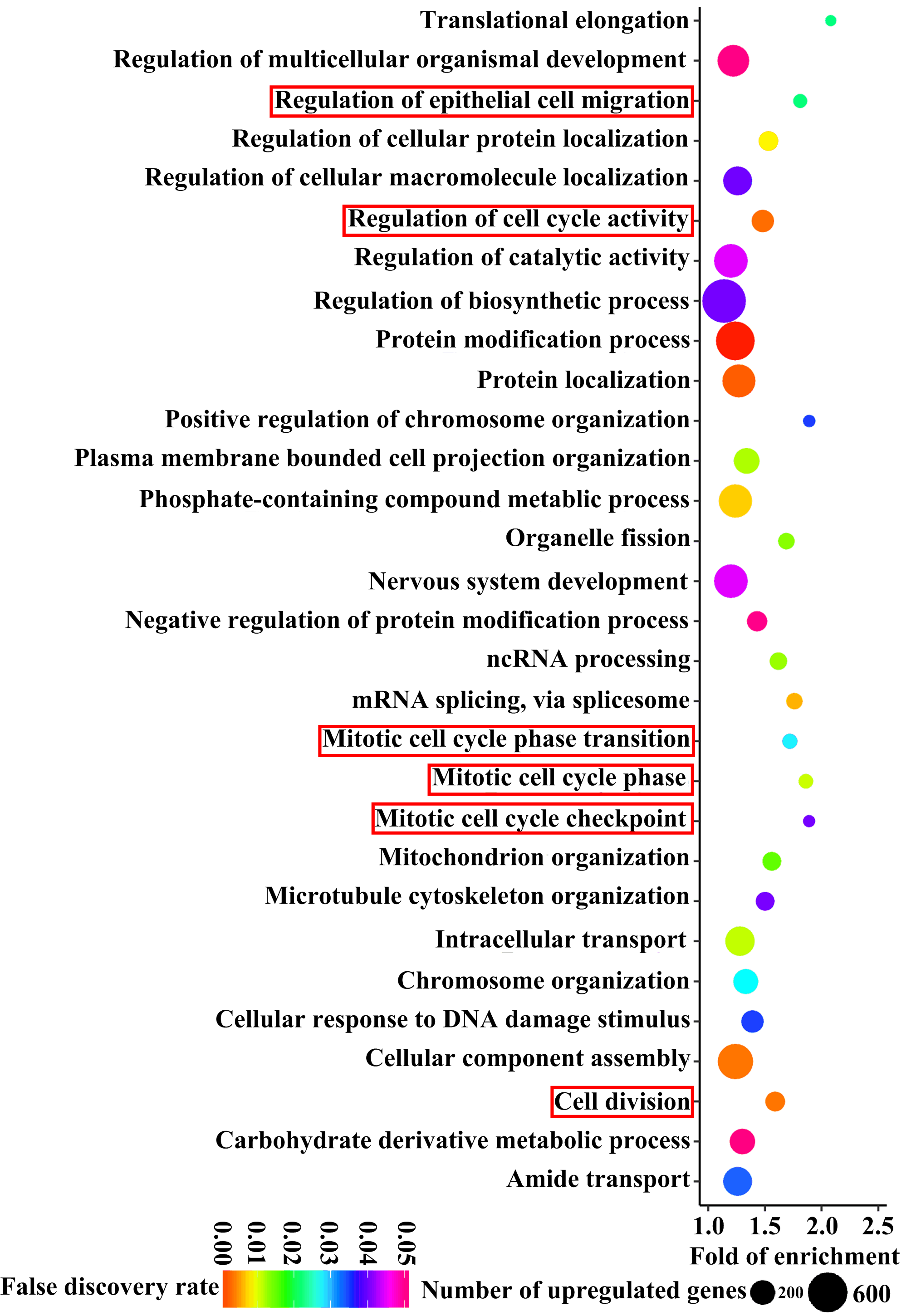


**Figure 4. Pathways associated with upregulated genes.** The bubblechart showed the pathways associated with upregulated genes (false discovery rate < 0.05). Y-axis label represented pathways, and X-axis label represented fold of enrichment. The sizes of the bubbles were proportional to the number of upregulated genes assigned to each pathway, and colors of the bubbles represented false discovery rate. Pathways associated with proliferation and migration were highlighted with red box.


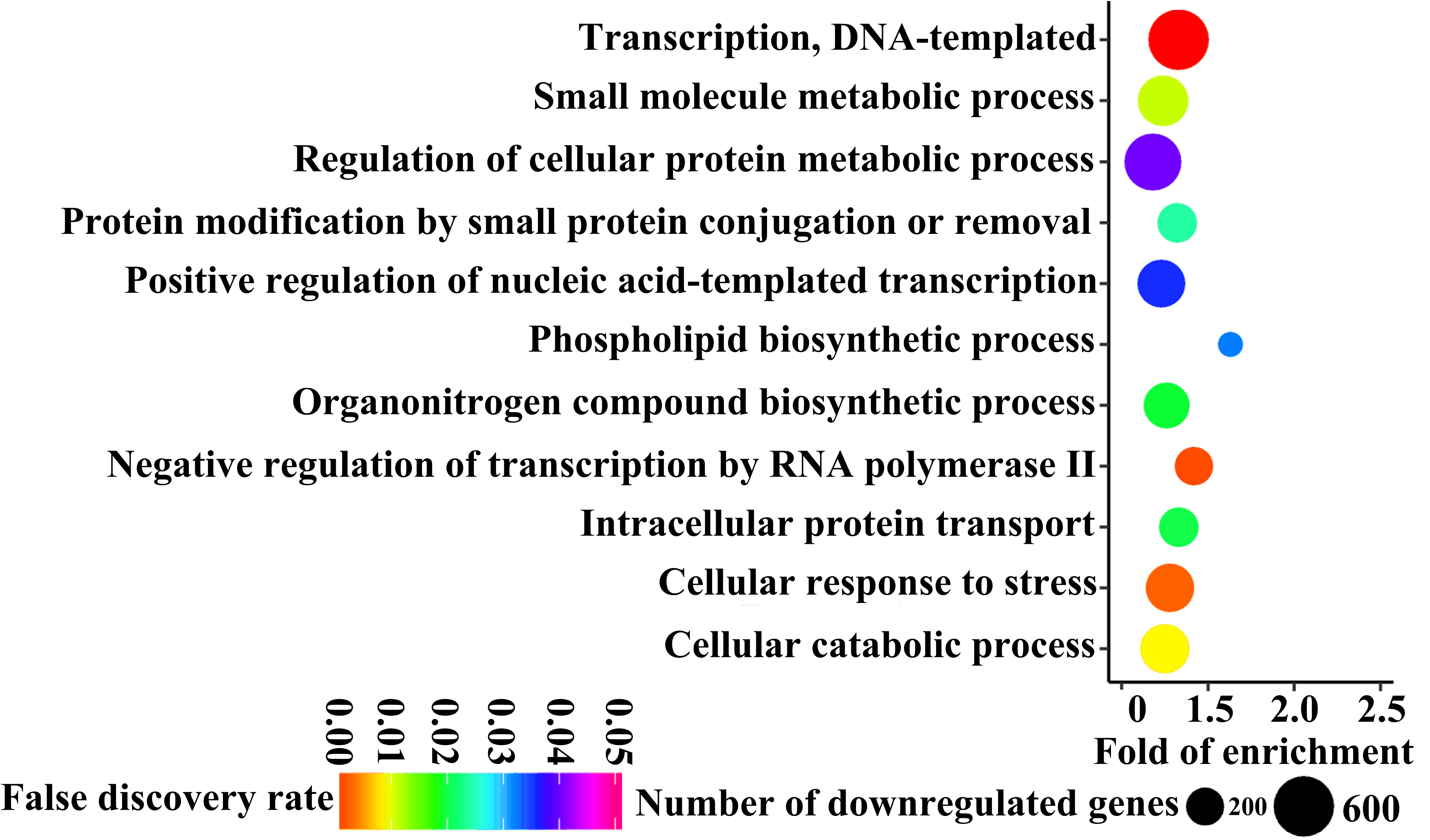


**Figure 5. Pathways associated with downregulated genes.** The bubblechart showed the pathways associated with downregulated mRNA (false discovery rate < 0.05). Y-axis label represented pathways, and X-axis label represented fold of enrichment. The sizes of the bubbles were proportional to the number of downregulated genes assigned to each pathway, and colors of the bubbles represented false discovery rate.

References

1. Wilhelm, K.; Happel, K.; Eelen, G.; Schoors, S.; Oellerich, M.F.; Lim, R.; Zimmermann, B.; Aspalter, I.M.; Franco, C.A.; Boettger, T.; Braun, T.; Fruttiger, M.; Rajewsky, K.; Keller, C.; Brüning, J.C.; Gerhardt, H.; Carmeliet, P.; Potente, M. FOXO1 couples metabolic activity and growth state in the vascular endothelium. Nature. 2016, 529, 216-220.
2. Jung, Y.A.; Lee, K.M.; Kim, M.K.; Jung, G.S.; Seo, Y.J.; Kim, H.S.; Cho, H.C.; Lee, K.U.; Park, K.G.; Lee, I.K. Forkhead transcription factor FoxO1 inhibits insulin- and transforming growth factor-beta-stimulated plasminogen activator inhibitor-1 expression. *Biochem Biophys Res Commun.* **2009,** 386, 757-761.
3. Skarra, D.V.; Arriola, D.J.; Benson, C.A.; Thackray, V.G. Forkhead box O1 is a repressor of basal and GnRH-induced Fshb transcription in gonadotropes. *Mol Endocrinol.* **2013,** 27, 1825-1839.
4. Mori, R.; Tanaka, K.; de Kerckhove, M.; Okamoto, M.; Kashiyama, K.; Tanaka, K.; Kim, S.; Kawata, T.; Komatsu, T.; Park, S.; Ikematsu, K.; Hirano, A.; Martin, P.; Shimokawa, I. Reduced FOXO1 expression accelerates skin wound healing and attenuates scarring. *Am J Pathol.* **2014,** 184, 2465-2479.
5. Rached, M.T.; Kode, A.; Silva, B.C.; Jung, D.Y.; Gray, S.; Ong, H.; Paik, J.H.; DePinho, R.A.; Kim, J.K.; Karsenty, G.; Kousteni, S. FoxO1 expression in osteoblasts regulates glucose homeostasis through regulation of osteocalcin in mice. *J Clin Invest.* **2010,** 120, 357-368.
6. Wu, Y.J.; Fang, Y.H.; Chi, H.C.; Chang, L.C.; Chung, S.Y.; Huang, W.C.; Wang, X.W.; Lee, K.W.; Chen, S.L. Insulin and LiCl synergistically rescue myogenic differentiation of FoxO1 over-expressed myoblasts. *PLoS One.* **2014,** 9, e88450.
7. Dong, X.C.; Copps, K.D.; Guo, S.; Li, Y.; Kollipara, R.; DePinho, R.A.; White, M.F. Inactivation of hepatic Foxo1 by insulin signaling is required for adaptive nutrient homeostasis and endocrine growth regulation. *Cell Metab.* **2008,** 8, 65-76.
8. Vasquez, Y.M.; Mazur, E.C.; Li, X.; Kommagani, R.; Jiang, L.; Chen, R.; Lanz, R.B.; Kovanci, E.; Gibbons, W.E.; DeMayo, F.J. FOXO1 is required for binding of PR on IRF4, novel transcriptional regulator of endometrial stromal decidualization. *Mol Endocrinol.* **2015,** 29, 421-433.
9. Kamei, Y.; Miura, S.; Suzuki, M.; Kai, Y.; Mizukami, J.; Taniguchi, T.; Mochida, K.; Hata, T.; Matsuda, J.; Aburatani, H.; Nishino, I.; Ezaki, O. Skeletal muscle FOXO1 (FKHR) transgenic mice have less skeletal muscle mass, down-regulated Type I (slow twitch/red muscle) fiber genes, and impaired glycemic control. *J Biol Chem.* **2004,** 279, 41114-41123.
10. Takano, M.; Lu, Z.; Goto, T.; Fusi, L.; Higham, J.; Francis, J.; Withey, A.; Hardt, J.; Cloke, B.; Stavropoulou, A.V.; Ishihara, O.; Lam, E.W.; Unterman, T.G.; Brosens, J.J.; Kim, J.J. Transcriptional cross talk between the forkhead transcription factor forkhead box O1A and the progesterone receptor coordinates cell cycle regulation and differentiation in human endometrial stromal cells. *Mol Endocrinol.* **2007,** 21, 2334-2349.
11. Lartey, L.J.; Werneck-de-Castro, J.P.; O-Sullivan, I.; Unterman, T.G.; Bianco, A.C. Coupling between Nutrient Availability and Thyroid Hormone Activation. *J Biol Chem.* **2015,** 290, 30551-30561.
12. Han, J.; Pan, X.Y.; Xu, Y.; Xiao, Y.; An, Y.; Tie, L.; Pan, Y.; Li, X.J. Curcumin induces autophagy to protect vascular endothelial cell survival from oxidative stress damage. *Autophagy.* **2012,** 8, 812-825.
13. Xie, Y.; Li, X.; Ge, J. Cyclophilin A-FoxO1 signaling pathway in endothelial cell apoptosis. *Cell Signal.* **2019,** 61, 57-65.
14. Hwa, Kim.S.; Das, A.; In, Choi.H.; Hoon, Kim.K.; Choul, Chai.J.; Ran, Choi.M.; Binas, B.; Sun, Park.K.; Seek, Lee.Y.; Jung, K.H.; Gyu, Chai.Y. Forkhead box O1 (FOXO1) controls the migratory response of Toll-like receptor (TLR3)-stimulated human mesenchymal stromal cells. *J Biol Chem.* **2019,** 294, 8424-8437.
15. Zeng, R.; Luo, D.X.; Li, H.P.; Zhang, Q.S.; Lei, S.S.; Chen, J.H. MicroRNA-135b alleviates MPP+-mediated Parkinson's disease in in vitro model through suppressing FoxO1-induced NLRP3 inflammasome and pyroptosis. *J Clin Neurosci.* **2019,** 65, 125-133.
16. Fabre, S.; Carrette, F.; Chen, J.; Lang, V.; Semichon, M.; Denoyelle, C.; Lazar, V.; Cagnard, N.; Dubart-Kupperschmitt, A.; Mangeney, M.; Fruman, D.A.; Bismuth, G. FOXO1 regulates L-Selectin and a network of human T cell homing molecules downstream of phosphatidylinositol 3-kinase. *J Immunol.* **2008,**181, 2980-2989.
17. Wang, H.; Zhang, Y.; Xia, F.; Zhang, W.; Chen, P.; Yang, G. Protective effect of silencing Stat1 on high glucose-induced podocytes injury via Forkhead transcription factor O1-regulated the oxidative stress response. *BMC Mol Cell Biol.* **2019,** 20, 27.
18. Altomonte, J.; Richter, A.; Harbaran, S.; Suriawinata, J.; Nakae, J.; Thung, S.N.; Meseck, M.; Accili, D.; Dong, H. Inhibition of Foxo1 function is associated with improved fasting glycemia in diabetic mice. *Am J Physiol Endocrinol Metab.* **2003,** 285, E718-E728.
19. Kim, D.H.; Ha, S.; Choi, Y.J.; Dong, H.H.; Yu, B.P.; Chung, H.Y. Altered FoxO1 and PPARγ interaction in age-related ER stress-induced hepatic steatosis. *Aging (Albany NY).* **2019,** 11, 4125-4144.
20. Matsuzaki, H.; Lee, S.; Maeda, M.; Kumagai-Takei, N.; Nishimura, Y.; Otsuki, T. FoxO1 regulates apoptosis induced by asbestos in the MT-2 human T-cell line. *J Immunotoxicol.* **2016,** 13, 620-627.
